# Primary central nervous system germ cell tumors in Central America and the Caribbean Region: an AHOPCA 20-year experience

**DOI:** 10.3389/fonc.2024.1393454

**Published:** 2024-07-05

**Authors:** Ana Verónica Girón, Jessica Blanco-Lopez, Patricia Calderon, Reyna Jiron, Estuardo Pineda, Margarita Montero, Yamel Lizardo, Ute Bartels, Diana S. Osorio

**Affiliations:** ^1^ Pediatric Oncology, Unidad Nacional de Oncología Pediátrica, Guatemala City, Guatemala; ^2^ Pediatric Oncology, Hospital Infantil Manuel de Jesús Rivera, Managua, Nicaragua; ^3^ Pediatric Oncology, Hospital Nacional de Niños Benjamín Bloom, San Salvador, El Salvador; ^4^ Pediatric Oncology, Hospital Infantil Dr. Robert Reid Cabral, Santo Domingo, Dominican Republic; ^5^ Hematology/Oncology, Hospital Infantil Regional Universitario Dr. Arturo Guillón, Santiago, Dominican Republic; ^6^ Department of Haematology/Oncology, The Hospital for Sick Children, Toronto, ON, Canada; ^7^ Department of Pediatrics, The University of Texas MD Anderson Cancer Center, Houston, TX, United States

**Keywords:** primary central nervous system germ cell tumors, chemotherapy, radiotherapy, survival rate, children, CNS tumors LMIC

## Abstract

**Background:**

Primary central nervous system germ cell tumors (GCT) are rare neoplasms in pediatrics. Treatment depends on the histological subtype and extent of the disease. Overall survival (OS) is above 90% for germinomas and 70%–80% for nongerminomatous GCT (NGGCT) in high-income countries (HIC) while data are usually lacking for patients in Low-Middle Income country (LMIC).

**Objective:**

This study aims to describe the experience of treating patients with CNS GCT in four of eight countries, members of the Asociación de Hemato-Oncología Pediátrica de Centro América (AHOPCA), and determine their 5-year OS.

**Design/methods:**

We conducted a retrospective chart review of patients treated for CNS GCT. Epidemiological and clinical characteristics, histology, treatment modalities, and outcomes were analyzed.

**Results:**

From 2001 to 2021, 48 patients were included: 22 from Guatemala, 18 from Nicaragua, three from the Dominican Republic, and five from El Salvador. Thirty-one (64.6%) were boys; the median age at diagnosis was 10.2 years (range: 1 to 17 years). Presenting symptoms were headaches (*n* = 24, 50%), visual disturbances (*n* = 17, 35.4%), vomiting (*n* = 12, 25%), nausea (*n* = 8, 16.7%), and diabetes insipidus (*n* = 7, 14.6%). Two patients with NGGCT presented with precocious puberty. Biopsy or tumor resection was performed in 38 cases (79.2%): 23 (88.4%) germinomas, 11 (78.6%) NGGCT, and four (50%) CNS GCT. Eight patients were diagnosed and treated based on CSF tumor marker elevation; four germinomas (BHCG 11.32–29.41 mUI/mL) and four NGGCT (BHCG 84.43–201.97 mUI/mL or positive AFP > 10 UI/mL). Tumor locations included suprasellar (*n* = 17, 35.4%), pineal (*n* = 13, 27.1%), thalamus/basal ganglia (*n* = 5, 10.4%), other (*n* = 12, 25%), and one bifocal. Four (8.3%) had metastatic disease, and six had positive CSF; staging data were incomplete in 25 patients (52%). Patients were treated with varied chemotherapy and radiotherapy modalities. Nine patients had incomplete data regarding treatment. Five-year OS was 65% (68% for germinoma, 50.6% for NGGCT, and 85.7% for unclassified GCT).

**Conclusions:**

Germinoma was the most common histology, and there was a male predominance. More than half of patients had incomplete staging data and treatment was variable across the region. OS is lower compared to HIC. Standardized treatment protocols will aid in adequate staging and treatment planning, prevent complications, and improve survival.

## Introduction

Central Nervous System Germ Cell tumors (CNS GCT) are a rare group of tumors in children (1). The 2021 WHO classification identifies GCT histology types as germinoma, teratoma (mature, immature, with somatic-type malignancy), yolk sac tumor, embryonal carcinoma, choriocarcinoma, and mixed germ cell tumors (2). The latter harbor different histology types. Usually, CNS GCT is classified into two groups, germinoma and nongerminomatous germ cell tumors (NGGCT; including all other histologies and mixed tumors) (2, 3). The histologic identification of these two groups and the extension of disease are fundamental for treatment planning and prognosis (4).

Incidence varies across different populations; North America reports < 3% of all CNS tumors, and parts of Asia report up to 16% in some regions (5–7).

For germinomas, 5-year survival rates are now reported above 90%, and for NGGCT, from 70% to more than 80%, in high-income countries (HIC) (8–11). In middle- and low-income countries (LMIC), survival varies, with reports of 5-year OS of 75%–88% for germinoma and 53%–75% for NGGCT (12, 13). In Latin America, Argentina reported 100% 5-year OS for localized germinoma and 75% for NGGCT. Brazil reported 100% 5-year OS for localized germinoma with chemotherapy, low-dose whole ventricular irradiation (WVI 18 Gy), and low-dose local boost (12 Gy) (14, 15).

Treatment modalities have changed through the years for the two GCT groups. Combination modalities with chemotherapy and reduced-field and reduced-dose radiotherapy in later studies demonstrated survival rates above 90% (10, 11). For localized germinoma showing complete response to chemotherapy, reducing whole ventricular (18 Gy) with boost to a total of 30 Gy or WVI (24 Gy) alone shows excellent OS, produced results similar to CSI radiotherapy alone, and is the most recent approach to treatment (15, 16). Bifocal germinoma is also treated as a localized disease and not metastatic, with excellent results (17). For metastatic germinoma, chemotherapy plus reduced-dose CSI radiotherapy and local boost to primary and metastatic lesions can also achieve survival rates similar to localized tumors. This approach has allowed to further reduce radiation dose and fields, and it is intended to reduce toxicity related to higher doses of radiotherapy (10, 11, 17, 18).

Improvement in survival for NGGCT has involved multimodality treatment that includes intense chemotherapy, CSI, and local radiotherapy with or without aggressive tumor resection (19–22). Recently, efforts have been made to stratify patients into different risk groups according to histology, tumor markers, and response to treatment and thus evaluate a possible dose reduction in radiotherapy (23).

The Asociación de Hemato-Oncología Pediátrica de Centro America (AHOPCA) group was formed in 1998 in collaboration with St. Jude Children’s Research Hospital and other institutions in North America and Europe in order to promote multidisciplinary care and education and to develop shared clinical guidelines applicable for the region. Participating members include institutions from Guatemala, El Salvador, Honduras, Nicaragua, Costa Rica, Panamá, the Dominican Republic, and Haiti. Since 2000, several treatment guidelines have been developed for different cancers, but not for brain tumors such as CNS GCT (24). At the moment, no data are published on the treatment and outcome for these tumors in the AHOPCA group. In this study, we conducted a retrospective review of 48 patients with CNS GCT treated in four participating countries across five institutions with the purpose of determining OS rates, identifying diagnostic and treatment challenges in our region, develope strategies to improve them.

## Patient and methods

### Patient selection

A retrospective review of patients diagnosed with CNS GCT was conducted from January 2001 to December 2021 in four participating countries across five institutions in Guatemala, Nicaragua, El Salvador, and the Dominican Republic (**Table 1**).

**Table 1:** Patient demographic and clinical characteristics.

	Total	
	N	%
**Total**	**48**	100
Sex		
Female	**17**	35.4
Male	**31**	64.6
Age		
Median [Q1–Q3]	**10.2 [8.0–12.8]**	
< 10 years	**20**	41.7
≥ 10 years	**28**	58.3
Symptoms		
Headache	**24**	50.0
Vision disturbances	**17**	35.4
Vomit	**12**	25.0
Nausea	**8**	16.7
DI	**7**	14.6
Precocious puberty	**2**	4.2
Other (e.g., seizures, hemiparesis)	**9**	18.8
Histopathology		
Germinoma	**26**	56.2
NGGCT	**14**	29.2
Not specified	**8**	16.7
Primary site		
Suprasellar	**17**	35.4
Pineal gland	**13**	27.1
Third ventricle	**4**	8.3
Thalamus	**4**	8.3
Posterior fossa	**4**	8.3
Bifocal	**1**	2.1
Basal ganglia	**1**	2.1
Not specified	**4**	8.3
Metastasis		
Yes	**4**	8.3
No	**35**	72.9
No data/Incomplete staging	**9**	18.8
Cerebro-spinal fluid		
Positive	**6**	12.5
Negative	**20**	41.7
Not performed/No data	**22**	45.8
Biopsy/Resection		
Yes	**38**	75.0
No	**9**	18.8
No data	**1**	6.2

Patient charts were reviewed, and data were collected on variables such as age, gender, tumor location, symptoms at diagnosis, histology, cerebral-spinal fluid (CSF) and serum tumor maker levels, the extent of resection if performed, chemotherapy and radiotherapy administered, follow-up time, delays in treatment, abandonment, and date of death.

### Diagnosis

The diagnosis was determined by neuroimaging, that in some centers was limited to computer tomography (CT) when access to magnetic resonance imaging (MRI) was limited, especially of the spine, for complete staging. Histological confirmation and/or serum and/or CSF markers from the time of diagnosis, when available. Pathology confirmation was performed by morphology alone with H&E due to the lack of immunohistochemistry across the region. When pathological diagnosis was not available, surgery was not performed, or tissue was not diagnostic, patients were diagnosed with levels of tumor markers. Guatemala was the only country where CSF tumor markers were able to be performed. They applied the same techniques and reagents used for serum markers. In the Dominican Republic, it can occasionally be done in private laboratories. Germinoma was considered a diagnosis if serum or CSF beta-human chorionic gonadotropin (βHCG) markers were between 5.3 and 50 mUI/L and negative for alpha-fetoprotein (AFP). Patients with serum and CSF βHCG values above 50 IU/L and/or positive for AFP (above 10 UI/mL) were considered NGGCT (25–27).

Metastatic disease was assessed with a postoperative spine MRI and CSF cytology, when available.

### Treatment

#### Surgery

In general, after a lesion was identified, surgery was attempted, typically at an adult center, by general neurosurgeons. Neurosurgeons often made the decision to refer patients for radiation therapy or to the referral pediatric oncology centers where patients received the remainder of their treatment. The extent of resection was generally determined by a postoperative image (MRI or CT) or by surgical report when available.

#### Chemotherapy

Different chemotherapy regimens were administered as the region did not have unified guidelines, and treatment strategies have also evolved over the last 20 years. The predominant chemotherapy regimens used were platinum-based, and regimens were not necessarily chosen based on GCT type. Since 2012, chemotherapy has been administered as per the International Society of Pediatric Oncology (SIOP) CNS GCT 96 protocol for germinoma and COG ACNS0122 for NGGCT in Guatemala. It was also used in other centers, but frequently treatment was decided case-by-case, based on consultations and case presentations with collaborative specialists and groups. In a group of patients from Nicaragua, the chemotherapy regimen is not specified (**Tables 2**, **3**).

**Table 2:** Treatment.

	Total		Germinoma		NGGCT		GCT not specified	
	N	%	N	%	N	%	N	%
**Total**	**48**	100	**26**	100	**14**	100	**8**	100
Overall therapy								
Surgery only	**3**	6.2	**0**	0	**3**	21.4	**0**	0
Radiotherapy only	**5**	10.4	**5**	19.2	**0**	0	**0**	0
Combined therapy (chemo + radiotherapy ± surgery)	**19**	39.6	**12**	46.1	**5**	35.7	**2**	25
Chemotherapy/No data on radiotherapy	**9**	18.7	**2**	7.7	**3**	21.4	**4**	50
Chemotherapy/No radiotherapy^a^	**11**	23	**8**	30.7	**3**	21.4	**1**	12.5
Treatment not specified	**1**	2.1	**0**	0	**0**	0	**1**	12.5
**Surgery**	**38**	79.2	23	88.4	11	78.6	4	50
Complete resection	**6**	12.5	**1**	2.1	**5**	10.4	**0**	0
Partial resection/Biopsy	**29**	60.4	**20**	41.6	**6**	12.5	**3**	6.25
Extent of surgery not specified	**3**	6.2	**2**	4.1	**0**	0	**1**	2.1
Chemotherapy								
Yes	**39**	81.2	**21**	80.8	**11**	78.6	**7**	87.5
No	**9**	18.8	**5**	19.2	**3**	21.4	**1**	12.5
Radiotherapy								
Yes	**24**	50.0	**17**	65.4	**5**	35.7	**2**	25.0
Focal only	**3**	6.2	**3**	11.5	**0**	0	**0**	0
Focal and CSI	**6**	12.5	**3**	11.5	**3**	21.4	**0**	0
Focal and WV	**6**	12.5	**5**	19.2	**1**	7.1	**0**	0
Focal and cranial	**1**	2.1	**1**	3.8	**0**	0	**0**	0
WV and CSI	**3**	6.2	**2**	7.7	**1**	7.1	**0**	0
WV only	**1**	2.1	**0**	0	**0**	0	**1**	12.5
Cranial only	**4**	8.3	**3**	11.5	**0**	0	**1**	12.5
Radiotherapy dose								
Total focal < 50 Gy	**5**	10.4	**4**	15.4	**1**	7.1	**0**	0
Total focal ≥ 50 Gy	**10**	20.8	**7**	26.9	**3**	21.4	**0**	0
Cranial only < 50 Gy	**1**	2.1	**1**	3.8	**0**	0	**0**	0
Cranial only ≥ 50 Gy	**3**	6.2	**3**	11.5	**0**	0	**0**	0
WVI ≤ 24 Gy	**8**	16.7	**6**	23.1	**2**	14.3	**0**	0
WVI > 24 Gy	**2**	4.2	**1**	3.8	**0**	0	**1**	12.5
CSI < 36 Gy	**3**	6.2	**2**	7.7	**1**	7.1	**0**	0
CSI = 36 Gy	**6**	12.5	**3**	11.5	**3**	21.4	**0**	0

a Eight patients died; two abandoned before radiation treatment.

WVI, whole ventricular irradiation; CSI, cranioespinal irradiation.

**Table 3:** Chemotherapy regimens.

Tumor type	Chemotherapy administered	*N*
**Germinoma**	Carboplatin/VP 16 (CNS GCT 96)	12
	No chemotherapy	5
	PEB	5
	Cisplatin/VP 16 with ifosfamide/VP 16	1
	Cisplatin/VP 16	1
	Carboplatin/Vincristine	1
	Chemotherapy regimen not specified	1
**NGGCT** (mixed and by markers)	Carboplatin/VP 16 alt ifosfamide/VP 16 (COG ACNS0122)	4
	PEB	2
	Chemotherapy regimen not specified	1
Germinoma with mature teratoma	Carboplatin/VP 16 × 4 (CNS GCT 96)	1
Immature teratoma	Cisplatin/VP 16	1
	PEB	1
Mature teratoma	None	3
	Chemotherapy regimen not specified	1
**GCT without histologic subclassification**		
Suprasellar	Chemotherapy regimen not specified	2
Pineal	PEB	2
	Chemotherapy regimen not specified	1
Thalamic	Chemotherapy regimen not specified	1
Posterior fossa	PEB	1
Unknown	None	1

VP 16, etoposide; PEB, cisplatin/etoposide/bleomycin.

#### Radiotherapy

Patients received a range of radiation therapy doses and fields independent of the GCT type. Treatment was based on their resources, availability, experience, and information at that time (**Table 3**). Similarly, radiation therapy doses and fields were decided following the SIOP CNS GCT 96 protocol for germinoma and COG ACNS0122 for NGGCT in a group of patients since 2012 or on a case-by-case basis after consultation with international experts in the field (**Table 2**).

### Statistical analysis

The outcome was estimated using the Kaplan–Meier method with Greenwood standard error (SE) and compared with the log-rank test if needed. The estimates included the following: abandonment-sensitive event-free survival (as-EFS), defined as the time from the beginning of treatment until the first event: death (related to treatment); treatment abandonment (if the patient was absent ≥ 4 consecutive weeks during therapy); progressive disease (PD); relapse; and second tumor. The overall survival (OS) was estimated as the time from the beginning of treatment until death (from any cause) or date of abandonment (assuming that patients who did not complete therapy succumbed to their disease).

## Results

This retrospective analysis examined data for 48 patients diagnosed with CNS GCT over a 20-year period (between January 2001 and December 2021). The majority of patients (22) were from Guatemala and received treatment at the Unidad Nacional de Oncología Pediátrica (UNOP). Eighteen patients were from Nicaragua and treated at the Hospital Escuela La Mascota. Five patients from El Salvador received treatment at the Hospital Nacional de Niños Benjamín Bloom, and two patients were from the Dominican Republic and treated at either the Hospital Infantil Regional Dr. Arturo Grullon or Hospital Infantil Robert Reid Cabral.

The median patients’ age was 10.2 years, ranging from 1 to 17 years. Boys made up 64.6% (28), while girls made up 35.4% (17).

The most frequent presenting symptoms were headache in 24 patients (50%), visual disturbances in 17 (35.4%), vomiting in 12 (25%), followed by nausea in eight patients (16.7%), and diabetes insipidus in seven patients (14.6%, five suprasellar tumors, one bifocal, and one pineal location). Two patients presented with precocious puberty; one tumor was located in the right thalamus, and the other was a suprasellar tumor. Both of these patients were diagnosed with an NGGCT. A 1-year 11-month-old patient presented with a regression of milestones. Other presenting symptoms included seizures, hemiparesis, conduct alterations, and ataxia.

The tumor was located in the suprasellar region in 17 patients (35.4%). Thirteen patients (27.1%) had a pineal tumor, and one patient (2.1%) had a bifocal tumor. Other tumor locations were the thalamus, third ventricle, and posterior fossa, with four (8.3%) patients in each of those locations, one located at the basal ganglia, and four patients without data. Of the four posterior fossa tumors, two were mature teratomas, one was an immature teratoma, and one GCT was not subclassified.

The tumor marker data available were from the initial diagnosis. Tumor marker level ranges included serum: βHCG 0.2–325.7 mIU/mL, with four patients having levels above 50 mIU/mL, and CSF: βHCG 11–201.97 mIU/mL, with three patients having levels above 50 mIU/mL. Serum AFP levels: 0–1,399 mIU/mL, and CSF AFP: 0–0.52 mIU/mL. Some results for both CSF and serum markers were reported only as negative. Serum tumor markers were done in 26 patients, and CSF tumor markers in 19 and all were performed at diagnosis. Four patients with positive BHCG levels below 50 mIU/mL were diagnosed as germinomas; three patients with BHCG levels greater than 50 mIU/mL and one with positive AFP were diagnosed as NGGCT; and this last patient had histologic confirmation with surgery after chemotherapy. Tumor marker data were not available for 22 patients (45.8%).

### Staging

Tumor staging was incomplete, with either CSF cytology (22 patients, 45.8%) and/or spinal MRI (25 patients, 52%) not done or data were not available. Three patients had neither CSF nor spinal MRI done (one mature teratoma, one immature teratoma, and one germinoma).

A total of four patients (8.33%) had metastatic ventricular lesions evidenced on brain imaging (all germinoma); six had CSF cytology positive for malignant cells, three of which had metastatic lesions on brain imaging.

**Figure 1:**
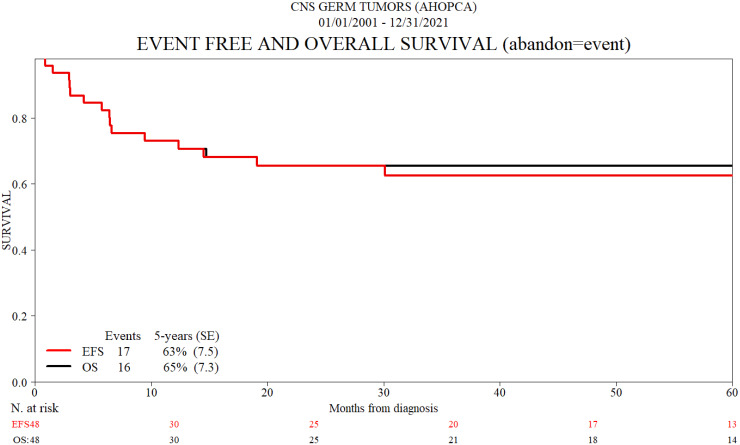
Event-free and overall survival (abandon = event).

Event survival (EFS) and OS at 5 years for this group of patients are 63% and 65%, respectively (**Figure 1**).

### Treatment

### Germinoma treatment and outcome

**Figure 2:**
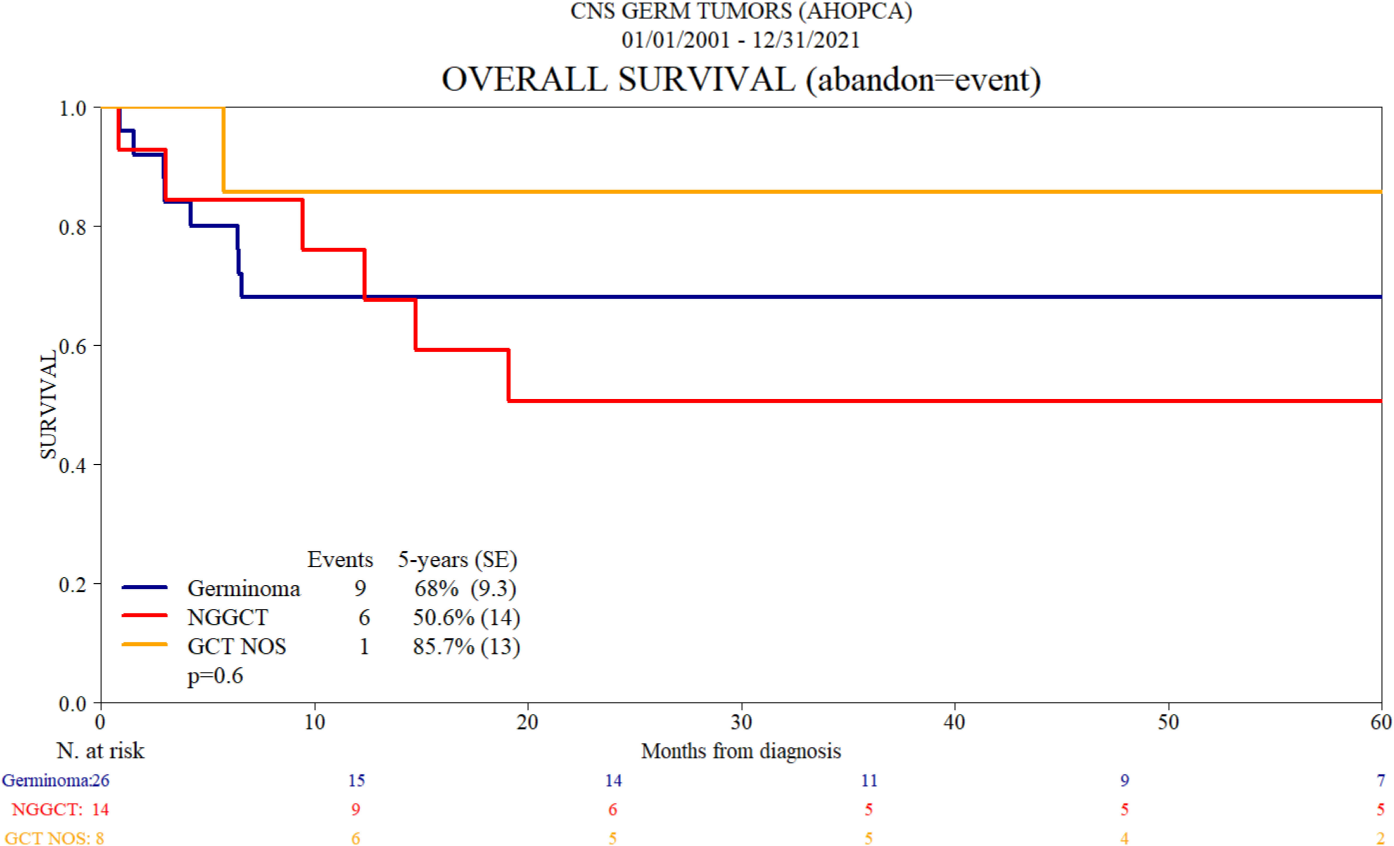
Overall survival (abandon = event).

In total, 26 patients (54.1%) were diagnosed with germinoma, four of those based on tumor marker results. Twenty-three patients had surgery: 11 had a biopsy, 10 had a partial resection, one had a total resection, and one had surgery with unknown results due to a lack of data. One patient who had a partial resection and was diagnosed with CNS GCT was later classified as having a germinoma with tumor makers. Monotherapy with radiation was used in five (19.2%) patients; three had focal radiation, and doses were 50 to 59.4 Gy total; one patient had CSI at 36 Gy and completed 54 Gy of focal radiation; and one had a total of 54.5 Gy focal and the same dose whole ventricular (WV). Twelve patients had combination therapy with radiation and chemotherapy, with a carboplatin/VP 16 (etoposide) regimen used, one in combination with ifosfamide/VP 16, usually four cycles. Other chemotherapy regimens were used in three patients, including one patient treated with PEB (cisplatin, etoposide, and bleomycin). Different radiation modalities were used; four patients received 24 Gy WV with a focal boost that completed 40 Gy in three patients and 50 Gy in one. Two patients received 24 Gy WV and 36 Gy CSI radiotherapy. Three patients received focal therapy with 50 to 60 Gy. Two patients received focal (50.4 Gy) and CSI (30.6 Gy), and one patient was treated with 30.6 Gy cranial radiation with a focal boost of 19.8 Gy. Six patients died during chemotherapy before radiotherapy, and one abandoned treatment and did not receive radiation therapy. Two patients received chemotherapy, but no data on radiation are available for these two patients. One patient who relapsed received rescue treatment and is currently alive. A total of eight patients died, and the causes of death include the following: intracranial hemorrhage after a car accident (one patient), sepsis due to *Candida tropicalis* (one patient), complications following VP shunt replacement with ventriculitis (one patient), complications from diabetes insipidus (one patient), complications after surgery (one patient), and disease progression (three patients). The 5-year OS rate for patients with germinoma was 68% (**Tables 2**, **4**; **Figure 2**).

**Table 4:** Last status.

	Germinoma		NGGCT		GCT nonclass		Total	
	N	%	N	%	N	%	N	%
**Enrolled**	**26**	100	**14**	100	**8**	100	**48**	100
Dead	**8**	30.8	**5**	35.7	**1**	12.5	**14**	29.2
Abandoned treatment	**1**	3.8	**1**	7.1	**0**	0.0	**2**	4.2
**Alive**	**17**	65.4	**7**	50.0	**7**	87.5	**31**	64.6
Lost to follow-up	**0**	0.0	**1**	7.1	**0**	0.0	**1**	2.1
Median follow-up time (months)	18.3		33.9		17.9			

### NGGCT treatment and outcome

Fourteen (29.1%) patients were diagnosed as NGGCT, four based on tumor marker results. Eleven patients underwent surgical resection, including one patient initially diagnosed with tumor markers after five cycles of chemotherapy who had a complete resection. Six patients had a partial resection. One patient with confirmed germinoma and teratoma histology received combined treatment with carboplatin/VP-16 for four cycles and radiation therapy (24 Gy WV and 40 Gy total focal). Two patients were treated with the PEB regimen. One received radiation therapy (25 Gy CSI and 50.5 Gy total focal) but died of progressive disease. Data on radiation treatment for the other patient are unavailable. One patient died of sepsis after one cycle of chemotherapy with carboplatin/VP-16 regimen. Two patients have no data on chemotherapy or radiation treatment and both died of progressive disease. Five patients had a complete resection, including three with mature teratoma; one abandoned treatment after five cycles of chemotherapy (carboplatin/VP-16 alternating with ifosfamide/VP-16); and one received combined treatment with the PEB regimen and radiation with 36 Gy CSI and 24 Gy WV. Of the three patients who had no surgery, two were treated with combined chemotherapy with carboplatin/VP-16 alternating with ifosfamide/VP-16 for six cycles total and radiation with 36 Gy CSI and 54 Gy total focal. Three patients with mature teratoma had no chemotherapy and appropriately did not proceed with radiation therapy. Five patients died; three had disease progression as the cause of death, one due to diabetes insipidus and complications from sepsis after three cycles of chemotherapy, and one after one cycle of chemotherapy due to sepsis. Five-year OS for NGGCT is 50.6% (**Tables 2**–**4**; **Figure 2**).

### Germ cell tumors without histologic subclassification treatment and outcome

Eight (16.6%) patients from Nicaragua were diagnosed with CNS GCT without histologic subclassification. None of the eight patients had tumor marker results in either CSF or serum. Four patients had surgery, two had a partial resection, one had a biopsy, and one had no data on the extent of surgery. All patients had chemotherapy, but the regimen was not detailed, and only one had data on receiving focal radiation at a dose of 59.4 Gy. Three patients had no surgery and received chemotherapy with regimens that were not detailed, and there were no data available on radiation therapy. One patient did not receive chemotherapy and has no data on surgery and radiotherapy. One patient died, but the cause of death is not reported. For this group, the 5-year OS survival is 85.5% (**Tables 2**–**4**; **Figure 2**).

### Radiation therapy availability

In Nicaragua, prior to 2019, radiation therapy was administered with a cobalt machine. Only more recently they have the ability to perform intensity-modulated radiation therapy (IMRT). IMRT has been available in Guatemala since 2009, in El Salvador since 2018, and is also available in the Dominican Republic, but we do not know the date they started using it.

## Discussion

This retrospective study helps us gain some insight into the treatment approach and outcomes for patients diagnosed with CNS GCT in Central America. We can also appreciate in this study how the resources can vary across the four Central American countries that form part of AHOPCA. They have similar challenges and limited resources as other LMICs within the LATAM region, such as the lack of pediatric neuro-oncology-trained subspecialists, pediatric neurosurgeons, multidisciplinary team meetings, access to resonance imaging prior to surgery, complete staging (spine MRI and CSF markers), and diagnostic pathology techniques beyond morphology (29). Although the patient characteristics and symptom presentation are expectedly similar to those of other HIC and MIC countries in the region, it is notable that the survival outcomes and treatment approach have great variability (12–15).

There are evident limitations to the diagnosis of patients with CNS GCT, as noted in our results. Diagnostic imaging and surgeries are usually performed based on CT imaging. Postoperative imaging can include MRIs, but they are not performed within a 24–72-h window after surgery. We do not have the data for this study, but the images for this review were usually performed a few weeks after surgery, when patients were transferred to the pediatric oncology units. The ability to determine the degree of leptomeningeal involvement or metastasis was difficult to ascertain in a very important percentage of patients since spine MRIs were generally not performed and the degree of metastases (presence of additional lesions or presence of leptomeningeal disease) was designated based on the brain imaging findings. Treatment planning and treatment response cannot be performed with incomplete and inadequate staging, as it is the standard of care to evaluate with a preoperative MRI at diagnosis and perform subsequent evaluations with routine resonance imaging and tumor markers (serum/CSF) (23, 27, 30, 31).

In Latin America, serum tumor markers are more commonly standardized and readily obtained compared to CSF tumor markers. Even though the reagents and laboratory techniques are similar for both serum and CSF markers, in Nicaragua, for example, they have been unsuccessful in obtaining CSF tumor markers, even after approaching privately funded laboratories. In Guatemala, the instructions on the labels of the reagents used do not specify they can be used for CSF. However, after further discussion with the chemical biologist and chief of the laboratory, they were able to overcome this barrier and provide CSF tumor marker results. Therefore, training for these laboratory techniques on CSF would be useful across the region for other countries to overcome this barrier.

Tumor values for diagnostic purposes were only available for a small group of patients since they were not routinely done before surgery. The cutoff level to define germinomas (βHCG < 50 mUI/mL) did not change over this period of time, even though there is evidence that germinoma can produce βHCG levels above 50 mUI/mL. This might have led to the overtreatment of patients with germinoma. The consensus on cutoff levels for tumor marker values varies around the world. The SIOP study defined germinoma with βHCG < 50 IU/L and NGGCT with serum or CSF AFP level of 25 ng/mL or higher and/or βHCG ≥ 50 IU/L (20, 27). The Children’s Oncology Group (COG) defined NGGCT with the level of 10 ng/mL for AFP and βHCG > 100 IU/L (23, 27). In a study in Brazil, cutoff levels for germinoma were undetectable levels of AFP and βHCG ≤ 200 mIU/L; NGGCT was defined as serum βHCG > 200 mIU/L and AFP > 5–10 ng/dL (9). Japanese studies have shown elevated βHCG levels above 200 mIU/L in germinomas and NGGCT with negative tumor markers; thus, they consider necessary histology confirmation as well as marker levels (28, 32, 33).

In this group of patients, tumor markers for follow-up were rarely taken, even though it is usually standard of care to evaluate treatment response and tumor recurrence (27, 28, 30, 31).

Local pathology still has limitations in that the diagnoses are carried out without immunohistochemical staining and are, to this day, based on histology and morphology alone. Therefore, it also puts into question which of the patients in this cohort may have a different diagnosis, particularly those patients who were tumor marker-negative.

Surgery was largely performed by neurosurgeons without pediatric subspecialty training. The region, as with other LMICs, has limitations in regards to trained neurosurgeons, instrumentation, and no data on complications (34). Since the majority of patients initially arrive at the neurosurgery unit and tumor markers are not done in these centers, a subgroup of patients with secreting GCT undergo surgery that can otherwise be spared, which can diminish complications after major surgery procedures (35, 36).

The chemotherapy regimens administered were mainly platinum-based, and the regimens varied among patients even from the same center. Some of the regimens used include cisplatin, bleomycin, and etoposide (PEB), cisplatin and etoposide, and carboplatin and etoposide with or without ifosfamide. Regimens sometimes were used independent of tumor histology and the regimen used in some patients is not specified. In some centers, since around 2012, as a result of the case-by-case consultation with international experts in pediatric neuro-oncology who provided timely feedback, chemotherapy regimens have been used as per the SIOP CNS GCT 96 protocol for germinoma and COG ACNS0122 for NGGCT for a number of patients (9, 23). Unfortunately, data on toxicity are not available, but one patient with germinoma died due to sepsis during chemotherapy treatment, and in the NGGCT group, two patients died also of sepsis after three and one cycles of chemotherapy. Challenges in supportive care are also mentioned in other publications that affect patients with GCT survival in LMICs (13).

Similar to chemotherapy administration, the approach to radiation treatment varied and was not always adapted to histology diagnosis, and no data are available on changes made based on tumor response to chemotherapy. IMRT has been available in Guatemala since 2009 and since 2019 in Nicaragua. Monotherapy with radiation was done in five patients with germinoma, with different doses and fields. Radiation alone, cranial, and CSI produce good outcomes in germinomas (8). As of 2012, many patients were treated with SIOP CNS GCT 96 protocol for germinoma and COG ACNS0122 for NGGCT (9, 23); others depended on recommendations after case-by-case consults. This collaboration allowed the reduction of dose and fields of radiotherapy, but new studies and protocols now approach treatment with an even greater reduction of radiation dose without compromising survival outcomes (14, 15). Therefore, there was not one single treatment approach across this region over this 20-year period and varied based on the treating physician’s criteria and/or resources available (**Table 3**).

We recognize that the diverse treatment approach demonstrated across the patients has very likely impacted our patient’s overall survival outcomes. Even though data on toxicity and complications are missing, two patients died due to diabetes insipidus and three due to sepsis, which reflects limitations in supporting treatment. As mentioned before, we share difficulties with other LMICs that can contribute to lower survival rates in these tumors, but we also have an example of a group in LATAM, such as Brazil, with a well-organized multidisciplinary team that has elaborated treatment protocols with excellent outcomes (15).

The AHOPCA group has continued collaboration with partners from HIC pediatric neuro-oncology experts. Additionally, the Latin America Brain Tumor Board (LATB) provides opportunities for expert neuro-oncology feedback in real-time and confirms the diagnoses of our patients with second pathology reviews and weekly individual case presentations (37). Also there is an effort from the St Jude Children’s Research Hospital, through the Global Alliance for Pediatric Neuro-Oncology (GAP-NO), to provide training and education to specialists in the region (38).

We hope that as future collaborations continue to occur across our region, we will be able to harmonize not only our treatment approaches but also our ability to share patient data in the hopes of improving the overall care and outcomes for children with brain tumors.

**Figure 3:**
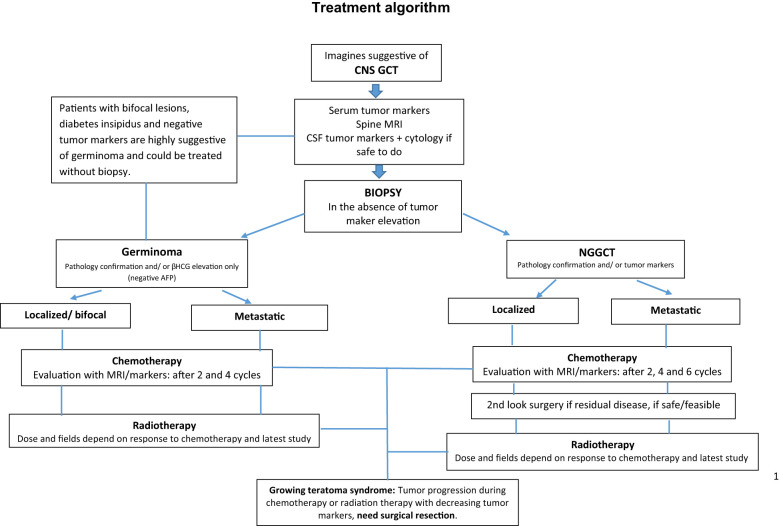
Treatment algorithm.

Additionally, we find this study demonstrates the need for unified, resource-based diagnostic and treatment guidelines for the region based on experts’ recommendations (**Figure 3**).

### Limitations

Our study lacks data due to several factors, such as data from patients treated many years ago that are no longer retrievable, challenges in obtaining data from outside institutions such as neurosurgery and radiotherapy units, and the lack of resources and support for data management and such personnel. Data on treatment toxicity and postsurgical complications is also not included for the same reason and should be a priority to be included in future studies.

Another limitation of our study was the lack of involvement of additional AHOPCA institutions across Central America, which were not able to share or obtain the data for this study and provide us with an even broader overview.

## Conclusions

This paper represents the first description of the overall treatment approach and outcome of patients with CNS GCT in Central America. Given the limitations described herein, it helps us understand the differences in OS compared to those of a HIC. We also believe that the early involvement of pediatric oncologists in the diagnosis of brain tumors will aid our local subspecialists in ensuring better treatment planning with more adequate staging and imaging. Furthermore, adapted chemotherapeutic regimens and standardized protocols may prevent complications and improve survival. Continued collaboration with the weekly LATB and GAP-NO is also of importance as the neuro-oncology field continues to advance in diagnostics and therapeutics.

## Data availability statement

The original contributions presented in the study are included in the article/supplementary material. Further inquiries can be directed to the corresponding authors.

## Ethics statement

Ethical approval was not required for the study involving humans in accordance with the local legislation and institutional requirements. Written informed consent to participate in this study was not required from the participants or the participants’ legal guardians/next of kin in accordance with the national legislation and the institutional requirements.

## Author contributions

AG: Supervision, Writing – review & editing, Writing – original draft, Project administration, Methodology, Investigation, Formal analysis, Data curation, Conceptualization. JB-L: Writing – review & editing, Software, Formal analysis. PC: Writing – review & editing, Data curation. RJ: Writing – review & editing, Data curation. EP: Writing – review & editing, Data curation. MM: Writing – review & editing, Data curation. YL: Data curation, Writing – review & editing. UB: Writing – review & editing, Supervision, Methodology, Conceptualization. DO: Writing – original draft, Supervision, Project administration, Methodology, Formal analysis, Conceptualization, Writing – review & editing, Data curation.
